# Association of Preoperative Coagulability With Incidence and Extent of Portal Vein Tumor Thrombus and Survival Outcomes in Hepatocellular Carcinoma After Hepatectomy: A Large-Scale, Multicenter Study

**DOI:** 10.3389/fonc.2021.697073

**Published:** 2021-07-28

**Authors:** Xiu-Ping Zhang, Teng-Fei Zhou, Jin-Kai Feng, Zi-Yang Sun, Zuo-Jun Zhen, Dong Zhou, Fan Zhang, Yi-Ren Hu, Cheng-Qian Zhong, Zhen-Hua Chen, Zong-Tao Chai, Kang Wang, Jie Shi, Wei-Xing Guo, Meng-Chao Wu, Wan Yee Lau, Shu-Qun Cheng

**Affiliations:** ^1^Department of Hepatic Surgery VI, Eastern Hepatobiliary Surgery Hospital, Second Military Medical University, Shanghai, China; ^2^Faculty of Hepato-Biliary-Pancreatic Surgery, The First Medical Center of Chinese People’s Liberation Army (PLA) General Hospital, Beijing, China; ^3^Department of Oncology, The No. 313 Hospital of PLA, Huludao, China; ^4^College of Basic Medical Sciences, Second Military Medical University, Shanghai, China; ^5^Department of Hepatobiliary Surgery, Foshan First People’s Hospital, Foshan, China; ^6^Department of Hepatobiliary Surgery, Fujian Cancer Hospital, Fuzhou, China; ^7^Department of Hepatobiliary Surgery, Affiliated Hospital of Binzhou Medical College, Binzhou, China; ^8^Department of General Surgery, Wenzhou People’s Hospital, Wenzhou, China; ^9^Department of Hepatobiliary Surgery, LongYan First Hospital, Affiliated to Fujian Medical University, Longyan, China; ^10^Department of General Surgery, Zhejiang Provincial Armed Police Corps Hospital, Hangzhou, China; ^11^Faculty of Medicine, The Chinese University of Hong Kong, Hong Kong, SAR China

**Keywords:** hepatocellular carcinoma, portal vein tumor thrombus, international normalized ratio, liver resection, survival outcomes

## Abstract

**Background:**

Occurrence of portal vein tumor thrombus (PVTT) worsens the outcomes of hepatocellular carcinoma (HCC) and imparts high economic burden on society. Patients with high risks of having hypercoagulation are more likely to experience thrombosis. Herein, we examined how preoperative international normalized ratio (INR) was related to the incidence and extent of PVTT, and associated with survival outcomes in HCC patients following R0 liver resection (LR).

**Methods:**

Patients with HCC and PVTT were enrolled from six major hospitals in China. The overall survival (OS) and recurrence-free survival (RFS) rates of individuals with different INR levels were assessed with Cox regression analysis as well as Kaplan-Meier method.

**Results:**

This study included 2207 HCC patients, among whom 1005 patients had concurrent PVTT. HCC patients in the Low INR group had a significantly higher incidence of PVTT and more extensive PVTT than the Normal and High INR groups (P<0.005). Of the 592 HCC subjects who had types I/II PVTT following R0 LR, there were 106 (17.9%), 342 (57.8%) and 144 (24.3%) patients in the High, Normal and Low INR groups, respectively. RFS and OS rates were markedly worse in patients in the Low INR group relative to those in the Normal and High INR groups (median RFS, 4.87 versus 10.77 versus 11.40 months, P<0.001; median OS, 6.30 versus 11.83 versus 12.67 months, P<0.001).

**Conclusion:**

Preoperative INR influenced the incidence and extent of PVTT in HCC. Particularly, patients with HCC and PVTT in the Low INR group had worse postoperative prognosis relative to the High and Normal INR groups.

## Introduction

Hepatocellular carcinoma (HCC) belongs to the category of top-five leading cancers which claims many lives worldwide. It is especially prevalent in Asia and Africa due to hepatitis B virus (HBV) infection (1, 2). So far, portal vein tumor thrombus (PVTT) is estimated to develop in 44% to 62.2% in advanced HCC patients (3, 4). For cases of untreated HCC accompanied by PVTT, the reported median survival time (MST) is in the range of 2.7 to 4.0 months (1). PVTT is the bottleneck which seriously limits the survival of advanced HCC patients.

Currently, the treatment approaches for HCC with PVTT remain tremendous divergent between the east and the west. According to the established practice guidelines for the management of HCC, the recommended drug for advanced HCC with PVTT is sorafenib (3, 5). Unfortunately, this drug only leads to an MST of about 6.5 months in most patients (6–8). Giannini et al. (9) suggested that the treatment of patients with advanced HCC should be personalized instead of oral sorafenib only. Abundant clinical evidence shows that liver resection (LR) with curative intent is superior to non-surgical interventions in selected cases of advanced HCC. This is particularly the case in patients with HCC with PVTT occurring in the first-order branch of the main portal vein (MPV) or above (4, 10–12). To ease the process of selecting the optimized treatment, an EHBH-PVTT scoring system has been constructed and applied in clinical decision-making (13). Additionally, great advances in perioperative management practices and surgical techniques have increased the safety of LR in HCC complicated with PVTT.

PVTT in HCC was once named as “malignant portal vein thrombosis” because the thrombosis was regarded as cancer-related. Previous studies reported that venous embolus/thrombosis was greatly affected by the risk of plasma hypercoagulability in patients with cancer, a phenomenon caused by the host’s response to cancerous cells or coagulation-promoting activity of cancer cells (14–16). High levels of plasma fibrinogen positively correlates with poor prognosis in various cancers (17). There is little evidence, however, to show the presence of a hypercoagulability state exists in HCC patients, especially in those with coexisting malignant portal vein thrombosis. International normalized ratio (INR) is a common laboratory indicator to determine the coagulation profile of an individual patient. The association of preoperative hypercoagulability with the prevalence and severity of PVTT as well as survival outcomes in patients with HCC remains unclear.

The present large-scale multicenter investigation explored the relationships between preoperative INR level and the prevalence rate and extent of PVTT, and survival outcomes of HCC post-LR.

## Materials and Methods

### Patients

This study was carried out at the Eastern Hepatobiliary Surgery Hospital (EHBH), Foshan First People’s Hospital (FFPH), Fujian Cancer Hospital (FCH), the Affiliated Hospital of Binzhou Medical College (AHBMC), LongYan First Hospital (LYFH), and Wenzhou People’s Hospital (WPH), between January 2010 and December 2017. Study approval was obtained from the Institutional Ethics Committees of participating hospitals. Patient enrollment and other procedures followed the ethical protocols of the 1975 Declaration of Helsinki (6th revision, 2008). Each patient signed voluntarily to participate before treatment. All patients gave permission to the publication of data obtained during the investigation. These participants were categorized into three categories using preoperative INR level, hypercoagulability (Low INR group), hypocoagulability (High INR group) and normal coagulability (Normal INR group).

### Diagnosis of PVTT

Eligible patients were identified based on postoperative histopathological examinations (4). The classification criteria proposed by Cheng were utilized to divide PVTT into four sub-types: I, segmental/sectoral arms of portal vein (PV); II, right or left PV; III, main portal vein (MPV); and IV, thrombus in MPV stretching towards the superior mesenteric vein (18, 19). For sub-type I and II PVTT, PVTT occurred in the first-order branch or above of the MPV, and they could be resected en bloc with the HCC in the liver.

### Enrollment of Participants

All participants were enrolled based on the following criteria. HCC patients were included if they were: (I) 18–70 years old; (II) complicated with PVTT; (III) Child-Pugh score in category A or selected B (scores ≤7); (IV) initially received R0 LR and no residual tumors were present in microscopic examination based on excised samples; (V) no prior preoperative anti-cancer treatments; (VI) no distant metastases or extrahepatic spread; (VII) no macroscopic vascular invasion; (VIII) absence of coagulation-related diseases. The exclusion criteria were: (I) patients with palliative-intend tumor resection; (II) a history of other malignancies; (III) preoperative anticoagulant therapy; (IV) incomplete clinical data or lost to follow-up.

### Surgical Protocols

The surgical procedures have been described in previous studies (19, 20). HCC patients with type I and II PVTT underwent curative LR en bloc with PVTT. For types III/IV PVTT, thrombectomy was carried out through an incision of the portal vein. The incision was closed after thorough flushing with normal saline after thrombectomy. The operation was performed under general anaesthesia using a right subcostal incision with a midline extension. Intraoperative ultrasonography was performed routinely to assess the number and size of lesions and the relationship of tumors to adjacent major vascular structures. The abdominal cavity was carefully searched for the extent of local disease, extrahepatic metastases and peritoneal seedings. Pringle’s maneuver was applied to occlude the blood inflow of the liver with cycles of 15 minutes clamp time/5 minutes unclamped time. For types III/IV PVTT, the occlusion was done distal to the PVTT. The clamp crushing method was used for liver parenchymal transection.

### Preoperative and Postoperative Investigations

Routine preoperative investigations included imaging and serological tests. Imaging examinations included abdominal ultrasonography, contrast-enhanced magnetic resonance imaging (MRI) and/or computed tomography (CT) scan of the abdomen, and plain radiography or non-contrast CT scan of the chest. All the radiological data were reviewed by two experienced radiologists. Routine preoperative laboratory investigations included complete blood counts, liver and renal function tests, hepatitis B and C serology, coagulation indexes, and serum tumor markers. The coagulability state of patients was reflected by the INR level, which was chosen from the latest examination within three days before surgery for data analysis. The criteria to define Low, Normal, and High INR were INR less than 0.80, INR in the range of 0.80–1.20, and INR higher than 1.20, respectively. Routine postoperative investigations included histopathological and immunohistochemical studies. The diagnosis of HCC and PVTT was only determined by histological examination of the resected surgical specimens. The histopathological evaluations were performed by two independent and experienced pathologists who were blinded to the study protocol.

### Follow-Up and End Points

Patients were regularly followed up once every 2 to 3 months for the first year and once every 6 months thereafter, until death or dropout from the follow-up program. In addition to history-taking and physical examinations, follow-ups were conducted using laboratory tests, abdominal ultrasonography, contrast-enhanced CT or MRI. The diagnosis of disease recurrence was based on typical imaging features with or without raised serum AFP levels. Once HCC recurrence was determined, patients were actively treated. Treatment for HCC recurrence included repeat LR, radiofrequency ablation, transarterial chemoembolization (TACE) or sorafenib, depending on the general condition, the liver functional reserve and the pattern of tumor recurrence of the patients.

The primary end points of this study were recurrence-free survival (RFS) and overall survival (OS). RFS was calculated from the date of LR to the date when HCC recurrence was first diagnosed or the date of last follow-up. OS was calculated from the date of LR to the date of patient’s death or the date of last follow-up.

### Statistical Analysis

Continuous variables were reported as means and standard deviations (SD) or as medians and interquartile ranges (IQR). Appropriate statistical tests (the independent samples T test or Mann-Whitney U test) were used. Categorical data were reported as counts and percentages, and compared using the Chi-square test or Fisher’s exact test. Univariate and multivariate analyses were conducted using the Cox proportional hazards regression model. Parameters with a P value less than 0.05 on univariate analysis were incorporated into multivariate analysis. Multivariate Cox regression analysis with a stepwise selection was performed to detect independent predictors of RFS and OS (the entry criteria for selection into the final multivariate model was P < 0.05). Survival curves of RFS and OS were generated using the Kaplan-Meier method and compared using the log-rank test. Median survival times and their corresponding 95% confidence intervals (CI) were reported. The data analyses were performed using the SPSS software version 24.0 (IBM Corporation, Armonk, NY, USA).

## Results

### Patient Characteristics

**Figure 1:**
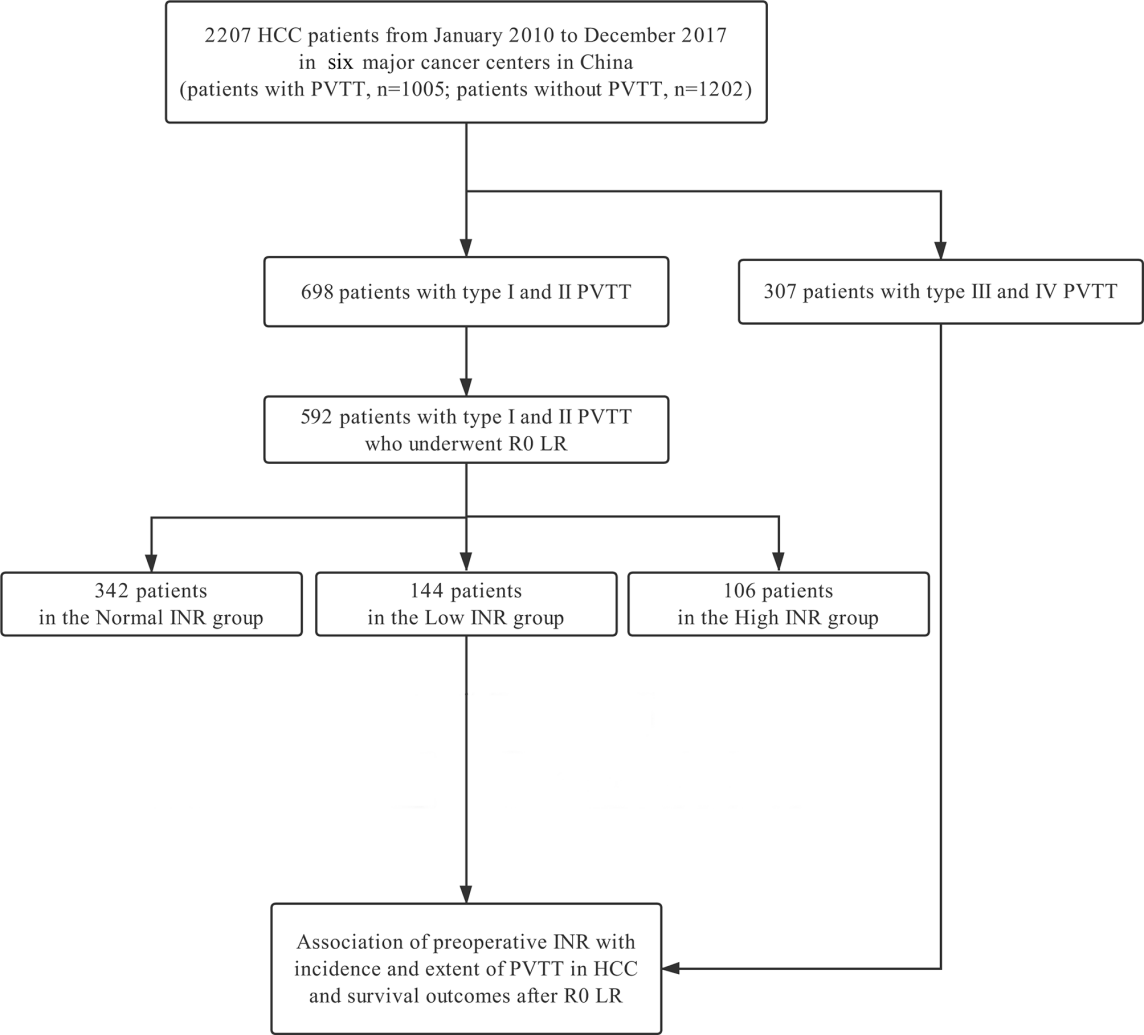
Flowchart of patient selection for the study. HCC, hepatocellular carcinoma; PVTT, portal vein tumor thrombus; LR, liver resection; INR, international normalized ratio.

This study included 2207 HCC patients at the six major cancer centers from January 2010 to December 2017 (**Figure 1**). These patients were divided into 2 groups according to whether they had PVTT (n = 1202 in the non-PVTT group; n = 1005 in the PVTT group). There were 698 patients with types I/II PVTT (592 underwent R0 LR), and 307 patients with types III/IV PVTT (86 underwent LR). The group of the 592 HCC patients with types I/II PVTT who underwent R0 LR was further divided into three groups according to preoperative INR levels (n = 106 in the High INR group; n = 342 in the Normal INR group; and n = 144 in the Low INR group). The clinicopathological features of HCC patients with types I/II PVTT who underwent R0 LR are shown in **Table 1**.

**Table 1:** The clinicopathological features of HCC patients with types I/II PVTT who underwent R0 LR (n=592).

Variables	INR Low (N=144)	INR Normal (N=342)	INR High (N=106)	P value
PVTT				
I	55 (38.19%)	113 (33.04%)	37 (34.91%)	0.551
II	89 (61.81%)	229 (66.96%)	69 (65.09%)	
Age (years)				
<50	73 (50.69%)	203 (59.36%)	61 (57.55%)	0.210
≥50	71 (49.31%)	139 (40.64%)	45 (42.45%)	
Sex				
Male	127 (88.19%)	316 (92.40%)	98 (92.45%)	0.292
Female	17 (11.81%)	26 (7.60%)	8 (7.55%)	
Child-Pugh				
A	143 (99.31%)	335 (97.95%)	103 (97.17%)	0.431
B	1 (0.69%)	7 (2.05%)	3 (2.83%)	
HBsAg				
Positive	127 (88.19%)	309 (90.35%)	95 (89.62%)	0.775
Negative	17 (11.81%)	33 (9.65%)	11 (10.38%)	
Ascites				
Yes	15 (10.42%)	36 (10.53%)	11 (10.38%)	0.999
No	129 (89.58%)	306 (89.47%)	95 (89.62%)	
No. of tumors				
Single	120 (83.33%)	298 (87.13%)	95 (89.62%)	0.325
Multiple	24 (16.67%)	44 (12.87%)	11 (10.38%)	
Satellite nodules				
Yes	130 (90.28%)	324 (94.74%)	95 (89.62%)	0.088
No	14 (9.72%)	18 (5.26%)	11 (10.38%)	
AFP (ng/mL)				
<400	46 (31.94%)	126 (36.84%)	39 (36.79%)	0.567
≥400	98 (68.06%)	216 (63.16%)	67 (63.21%)	
Lymph node invasion				
Yes	20 (13.89%)	53 (15.50%)	14 (13.21%)	0.804
No	124 (86.11%)	289 (84.50%)	92 (86.79%)	
Tumor diameter (cm)				
<5	32 (22.22%)	58 (16.96%)	20 (18.87%)	0.394
≥5	112 (77.78%)	284 (83.04%)	86 (81.13%)	
Tumor encapsulation				
Yes	43 (29.86%)	149 (43.57%)	55 (51.89%)	**0.001**
No	101 (70.14%)	193 (56.43%)	51 (48.11%)	
Cirrhosis				
Yes	91 (63.19%)	252 (73.68%)	84 (79.25%)	**0.012**
No	53 (36.81%)	90 (26.32%)	22 (20.75%)	
TBIL (μmol/L)				
<17.1	97 (67.36%)	249 (72.81%)	69 (65.09%)	0.226
≥17.1	47 (32.64%)	93 (27.19%)	37 (34.91%)	
DBIL(μmol/L)				
<6.8	90 (62.50%)	231 (67.54%)	66 (62.26%)	0.430
≥6.8	54 (37.50%)	111 (32.46%)	40 (37.74%)	
ALB (g/L)	41.5 (40.8–42.1)	41.7 (41.3–42.1)	39.8 (38.2–41.5)	**0.004**
ALT (U/L)	55.2 (46.4–64.1)	53.8 (49.5–58.1)	53.2 (47.2–59.2)	0.921
AST (U/L)	60.3 (52.5–68.0)	56.6 (52.5–60.7)	61.8 (54.4–69.2)	0.428
GGT (U/L)	174.0 (149.6–198.2)	169.2 (153.5–184.8)	172.0 (141.8–202.2)	0.946
ALP (U/L)	121.4 (113.5–129.4)	121.1 (114.6–127.5)	121.8 (112.0–131.5)	0.993
PLT (×10^9^/L)	171.8 (159.6–184.1)	166.9 (159.1–174.8)	158.3 (144.1–172.6)	0.361
CA199 (U/mL)	34.7 (27.6–41.9)	35.2 (28.1–42.2)	44.5 (24.2–64.7)	0.461

PVTT, portal vein tumor thrombus; HBsAg, hepatitis B surface antigen; AFP, α-fetoprotein; TBIL, total bilirubin; DBIL, direct bilirubin; ALB, albumin; ALT, alanine aminotransferase; AST, aspartate aminotransferase; GGT, γ-glutamyltransferase; ALP, alkaline phosphatase; PLT, platelet; CA199, carbohydrate antigen 19-9. P values in bold denote statistically significant difference.

### Association of Preoperative INR With Incidence and Extent of PVTT in HCC Patients

**Figure 2:**
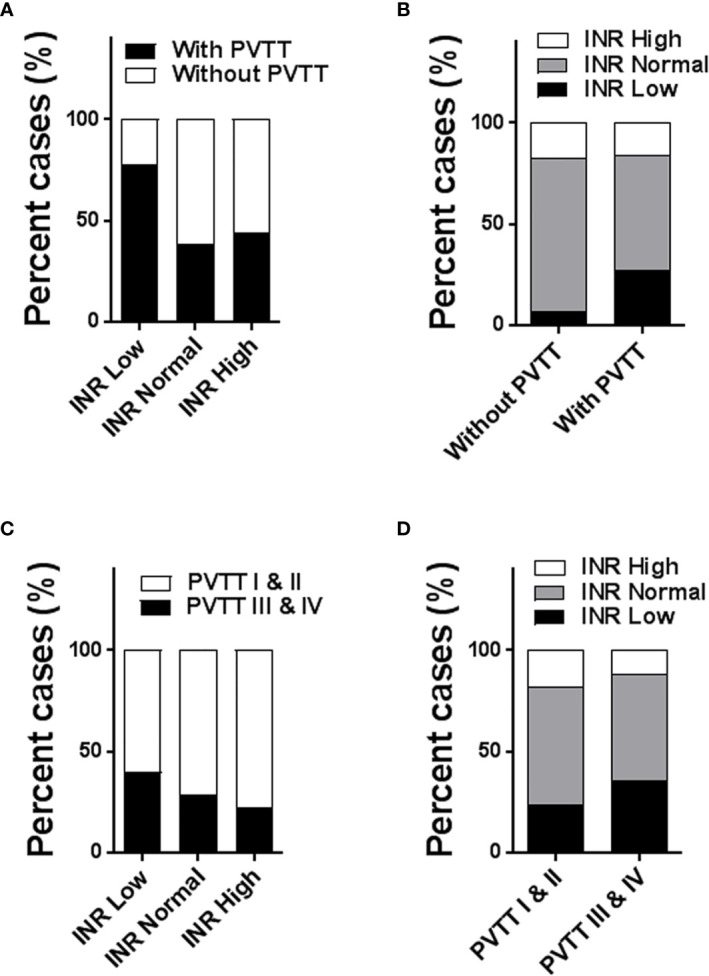
Histogram to show the association of preoperative INR level with incidence and extent of PVTT (1005 patients with PVTT *vs* 1202 patients without PVTT; 698 patients with types I/II PVTT *vs* 307 patients with types III/IV PVTT). The incidence rates of PVTT among the Low, Normal, and High INR groups **(A)**; the distributions of INR level between patients with and without PVTT **(B)**; the incidence rates of types I/II and types III/IV PVTT among the Low, Normal, and High INR groups **(C)**; the distributions of INR level between patients with types I/II PVTT and types III/IV PVTT **(D)**. PVTT, portal vein tumor thrombus; INR, international normalized ratio.

The incidence rate of PVTT was higher in HCC patients with a low INR level compared to those with a normal or high INR level (77.4% *vs* 38.2% *vs* 44.3% of patients with PVTT, in the Low, Normal and High INR groups, respectively, P<0.001) (**Figure 2A**). Of the 1202 patients who did not have PVTT, 80 patients (6.7%) were in the Low INR group, 916 patients (76.2%) in the Normal INR group, and 206 patients (17.1%) in the High INR group, respectively. Of the 1005 patients who had PVTT, 164 patients (16.3%), 567 patients (56.4%), and 274 patients (27.3%) were in the High, Normal, and Low INR groups, respectively. Notably, a low preoperative INR level was more likely to be observed in HCC patients associated with PVTT compared to those without PVTT (P<0.001) (**Figure 2B**).

Patients who had hypercoagulability in the Low INR group had more extensive PVTT (type III and IV PVTT *vs* type I and II PVTT) in comparison to the Normal and High INR groups (39.8% *vs* 28.4% *vs* 22.6% with type III and IV PVTT, 60.2% *vs* 71.6% *vs* 77.4% with type I and II PVTT in the Low, Normal and High INR groups, respectively, P<0.005) (**Figure 2C**). In other words, a significantly lower proportion of subjects with type I and II PVTT had hypercoagulability than those having type III and IV PVTT (23.6% *vs* 35.5% in the Low INR group, 58.2% *vs* 52.4% in the Normal INR group, and 18.2% *vs* 12.1% in the High INR group, respectively, P<0.005) (**Figure 2D**).

Taken together, HCC patients with hypercoagulability reflected by a low INR level had a higher incidence of PVTT and more extensive PVTT.

### Independent Prognostic Factors of Survival Outcomes

Univariate and multivariate Cox regression analyses conducted on HCC patients with type I and II PVTT after R0 LR demonstrated that type of PVTT (P=0.001), INR level (P<0.001), AFP (P<0.001), tumor diameter (P=0.011), and direct bilirubin (P<0.001) to be independent predictors of OS (**Table 2**). Type of PVTT (P=0.004), INR level (P<0.001), AFP (P<0.001), tumor encapsulation (P<0.001), and aspartate aminotransferase (P=0.011) were independent predictors of RFS (**Table 3**).

**Table 2:** Univariate and multivariate analysis of overall survival of 592 patients with types I/ II PVTT who underwent R0 LR.

Characteristics		OS (Univariate analysis)				OS (Multivariate analysis)			
		HR	95% CI		P value	HR	95% CI		P value
PVTT	II *vs* I	1.450	1.192	1.764	<0.001	1.388	1.137	1.693	0.001
INR	Normal *vs* Low	0.602	0.517	0.700	<0.001	0.595	0.511	0.694	<0.001
Age (years)	<50 *vs* ≥50	0.951	0.793	1.141	0.591				
Sex	Male *vs* Female	0.976	0.714	1.336	0.881				
Child-Pugh	A *vs* B	0.691	0.327	1.461	0.333				
HBsAg	Positive *vs* Negative	1.008	0.748	1.358	0.960				
Ascites	Yes *vs* No	1.400	1.056	1.855	0.019				
No. of tumors	Single *vs* Multiple	0.988	0.756	1.291	0.930				
Satellite nodules	Yes *vs* No	1.172	0.830	1.654	0.367				
AFP (ng/mL)	≥400 *vs* <400	2.288	1.542	3.404	<0.001	2.476	1.653	3.719	<0.001
Lymph node invasion	Yes *vs* No	1.080	0.843	1.384	0.543				
Tumor diameter (cm)	< 5 *vs* ≥ 5	0.778	0.621	0.973	0.028	0.744	0.592	0.934	0.011
Tumor encapsulation	Yes *vs* No	0.975	0.878	1.082	0.633				
Cirrhosis	Yes *vs* No	1.262	0.828	1.896	0.277				
TBIL (μmol/L)	≥17.1 *vs* <17.1	1.194	0.983	1.450	0.074				
DBIL (μmol/L)	≥6.8 *vs* <6.8	1.380	1.145	1.663	0.001	1.796	1.371	2.353	<0.001
ALB (g/L)		0.996	0.974	1.018	0.723				
ALT (U/L)		0.999	0.997	1.001	0.289				
AST (U/L)		1.002	1.000	1.004	0.041				
GGT (U/L)		1.000	0.999	1.001	0.925				
ALP (U/L)		1.002	1.000	1.003	0.027				
PLT (×10^9^/L)		1.000	0.999	1.001	0.928				
CA199 (U/mL)		1.000	0.999	1.002	0.644				

PVTT, portal vein tumor thrombus; INR, international normalized ratio; HBsAg, hepatitis B surface antigen; AFP, α-fetoprotein; TBIL, total bilirubin; DBIL, direct bilirubin; ALB, albumin; ALT, alanine aminotransferase; AST, aspartate aminotransferase; GGT, γ-glutamyltransferase; ALP, alkaline phosphatase; PLT, platelet; CA199, carbohydrate antigen 19-9.

**Table 3:** Univariate and multivariate analysis of recurrence-free survival of 592 patients with types I/II PVTT who underwent R0 LR.

Characteristics		RFS (Univariate analysis)				RFS (Multivariate analysis)			
		HR	95% CI		P value	HR	95% CI		P value
PVTT	II *vs* I	1.406	1.146	1.726	0.001	1.351	1.100	1.660	0.004
INR	Normal *vs* Low	0.697	0.594	0.818	<0.001	0.748	0.637	0.878	<0.001
Age (years)	<50 *vs* ≥50	0.989	0.813	1.203	0.910				
Sex	Male *vs* Female	0.947	0.673	1.334	0.757				
Child-Pugh	A *vs* B	0.673	0.300	1.508	0.336				
HBsAg	Positive *vs* Negative	1.088	0.788	1.503	0.609				
Ascites	Yes *vs* No	1.221	0.895	1.665	0.208				
No. of tumors	Single *vs* Multiple	0.829	0.615	1.119	0.220				
Satellite Nodules	Yes *vs* No	1.516	1.011	2.273	0.044				
AFP (ng/mL)	≥400 *vs* <400	2.566	1.673	3.968	<0.001	2.534	1.631	3.938	<0.001
Lymph node invasion	Yes *vs* No	1.220	0.938	1.586	0.138				
Tumor diameter (cm)	<5 *vs* ≥5	0.932	0.723	1.202	0.589				
Tumor encapsulation	Yes *vs* No	0.795	0.708	0.893	<0.001	0.803	0.715	0.902	<0.001
Cirrhosis	Yes *vs* No	1.184	0.953	1.470	0.127				
TBIL (μmol/L)	≥17.1 *vs* <17.1	1.087	0.878	1.345	0.445				
DBIL (μmol/L)	≥6.8 *vs* <6.8	1.038	0.844	1.276	0.724				
ALB (g/L)		1.016	0.991	1.042	0.214				
ALT (U/L)		1.000	0.998	1.002	0.991				
AST (U/L)		1.003	1.000	1.005	0.021	1.003	1.001	1.005	0.011
GGT (U/L)		1.000	0.999	1.001	0.719				
ALP (U/L)		1.001	0.999	1.002	0.394				
PLT (×10^9^/L)		1.001	0.999	1.002	0.292				
CA199 (U/mL)		0.999	0.997	1.001	0.181				

PVTT, portal vein tumor thrombus; INR, international normalized ratio; HBsAg, hepatitis B surface antigen; AFP, α-fetoprotein; TBIL, total bilirubin; DBIL, direct bilirubin; ALB, albumin; ALT, alanine aminotransferase; AST, aspartate aminotransferase; GGT, γ-glutamyltransferase; ALP, alkaline phosphatase; PLT, platelet; CA199, carbohydrate antigen 19-9.

### Survival Analysis in 592 HCC Patients With Types I/II PVTT Among the Three Groups With Different INR Levels

**Figure 3:**
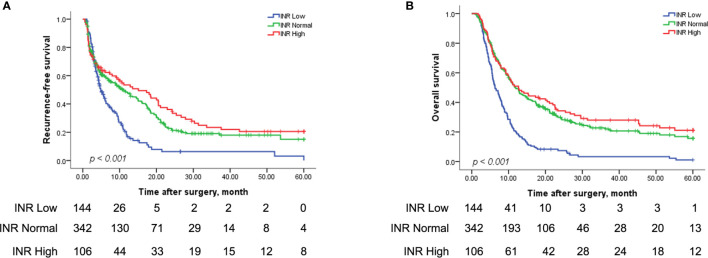
Kaplan-Meier analysis for the RFS and OS rates in HCC patients with types I/II PVTT after R0 LR among the Low, Normal, and High INR groups. RFS for patients among the Low, Normal, and High INR groups (144 patients *vs* 342 patients *vs* 106 patients) after R0 LR **(A)** (P < 0.001); OS for patients among the Low, Normal, and High INR groups (144 patients *vs* 342 patients *vs* 106 patients) after R0 LR **(B)** (P < 0.001). INR, international normalized ratio.

The three groups of subjects with type I and II PVTT with various INR levels had markedly different RFS and OS rates (both P<0.001) (**Figure 3**). **Figure 3A** illustrated that the 1-, 3-, and 5-year RFS rates were significantly poorer in the Low INR group relative to those in the Normal and High INR groups (1 year, 16.9% *vs* 48.3% *vs* 53.5%; 3 years, 6.3% *vs* 19.1% *vs* 23.4%; 5 years, 0% *vs* 15.0% *vs* 20.5%; median RFS, 4.87 *vs* 10.77 *vs* 11.40 months, P<0.001). Similarly, **Figure 3B** suggested that the 1-, 3-, and 5-year OS rates of the Low INR group were markedly worse than the Normal and High INR groups (1 year, 20.1% *vs* 49.6% *vs* 50.9%; 3 years, 3.3% *vs* 22.7% *vs* 28.0%; 5 years, 1.1% *vs* 15.7% *vs* 21.2%; median OS, 6.30 *vs* 11.83 *vs* 12.67 months, P<0.001). Nevertheless, the RFS and OS rates between the Normal and High INR groups were not statistically significantly different.

### Subgroup Analysis on Survival Outcomes for Patients With Types I/II PVTT in the Different Groups

The RFS was significantly worse in cases with low levels of INR compared to those with normal and high INR levels in type I PVTT (1 year, 19.4% *vs* 60.4% *vs* 68.6%; 3 years, 0% *vs* 19.4% *vs* 30.9%; 5 years, 0% *vs* 12.9% *vs* 30.9%, median RFS, 5.70 *vs* 16.07 months *vs* 19.50 months; P <0.001, **Supplementary Figure 1A**). Similarly, patients with type I PVTT with low levels of INR exhibited significantly poorer 1-, 3-, and 5-year OS rates than those with normal and high levels of INR (1 year, 20.0% *vs* 69.8% *vs* 70.3%; 3 years, 0% *vs* 38.6% *vs* 39.2%; 5 years, 0% *vs* 25.0% *vs* 28.0%, median OS, 5.80 *vs* 21.1 months *vs* 20.5 months; P <0.001, **Supplementary Figure 1B**). Equivalent results were obtained for survival of these three groups of HCC patients with type II PVTT after R0 LR (for RFS rates: 1 year, 15.1% *vs* 41.9% *vs* 45.3%; 3 years, 6.5% *vs* 19.9% *vs* 18.8%; 5 years, 0% *vs* 18.0% *vs* 13.4%, median RFS, 4.60 *vs* 6.80 months *vs* 7.97 months, P <0.001, **Supplementary Figure 1C**; for OS rates: 1 year, 20.2% *vs* 39.5% *vs* 40.6%; 3 years, 4.3% *vs* 16.3% *vs* 22.5%; 5 years, 1.4% *vs* 11.8% *vs* 17.9%, median OS, 6.67 *vs* 9.23 months *vs* 9.97 months; P <0.001, **Supplementary Figure 1D**).

## Discussion

The presence of PVTT is regarded as one of the most vital risk factors for HCC patients and always leads to unfavorable prognosis (9, 10). The available treatment for HCC complicated with PVTT is limited and the therapeutic effectiveness is not satisfactory. Owing to the great development of surgical techniques and perioperative management approaches, R0 LR operated for patients with HCC and type I and II PVTT becomes safe, and the perioperative morbidity and mortality rates have much decreased (4, 21). However, the related clinicopathological factors of PVTT occurrence largely remain to be elucidated.

To our knowledge, there is no study which focuses on determining the association between coagulation status and the incidence and extent of PVTT and survival outcomes in HCC patients. This study preliminarily investigated such an association based on a large-scale and multicenter data.

Macroscopic invasion of the main portal vein or its branches is commonly categorized to be advanced HCC (22, 23). In fact, little is known about the factors related to the occurrence and development of PVTT. A previous study reported by our laboratory revealed that HBV infection promotes PVTT development in HCC by activating and modifying the TGF-β-miR-34α-CCL22 pathway, which forms an immune-subversive micro-environment that enhances the colonization of cancerous cells in the portal vein (24). In addition, platelets were also demonstrated to regulate HCC progression and metastasis, and associated with the long-term prognosis of PVTT patients. Another study reported by our team demonstrated that preoperative thrombocytopenia independently predicted prolonged RFS and OS of individuals with HCC complicated with PVTT following hepatectomy, and high platelet counts were associated with a high rate of intrahepatic metastasis. All these add to the gathering evidence that anti-platelet drugs, such as aspirin, are potentially useful treatments for HCC with PVTT (25). A study by Gon et al. (26) indicated that liver fibrosis, AFP and extent of PVTT were independent risk factors of rapid progression of PVTT, whereas des-gamma-carboxy prothrombin (DCP), extent of PVTT and liver fibrosis were independent prognostic factors in HCC patients with PVTT.

Up to now, there is still very little evidence to demonstrate the possible relationship between coagulability state and development of PVTT in HCC patients, and the interplay between HCC cells and coagulation homeostasis is not entirely understood (27, 28). In this large-scale multicenter study, a considerably higher percentage of HCC patients with PVTT had lower INR levels relative to HCC patients without PVTT. These results indicated a close relationship between hypercoagulability and the incidence of PVTT. On the other hand, PVTT as foreign tumor masses can further promote the formation of hypercoagulability in the portal venous system. This study also showed HCC patients with types III/IV PVTT to have a significantly higher proportion of a low INR level (hypercoagulability) than those with types I/II PVTT, which suggested the potential association between coagulability status and the extent of PVTT. Hypercoagulability can increase the rate of progression of PVTT from a segmental/sectoral branch (Cheng’s type I) or right/left portal vein (Cheng’s type II) to the main portal vein (Cheng’s type III) or the superior mesenteric vein (Cheng’s type IV).

The association between preoperative coagulation state and outcomes of HCC subjects with PVTT following R0 LR has seldom been studied. In our study, HCC patients with PVTT with hypercoagulability (low INR level) had a worse prognosis compared to those with normal coagulability (normal INR level) or hypocoagulability (high INR level) after R0 LR. Patients with hypocoagulability in the High INR group exhibited comparable survival as patients with normal coagulability in the Normal INR group. On subgroup analysis, type I or type II PVTT patients with low levels of INR had poorer prognosis than those with normal or high levels of INR. On multivariate analysis, the preoperative INR level was an independent prognosis predictive factor of OS and RFS outcomes in the study population. The liver is an organ which synthesizes most coagulation factors and regulatory proteins, which play a central role in the coagulation regulation and hemostatic control. A review revealed that derangement in liver function can result in thrombotic complications, such as PVTT (15). Multiple and inter-connected mechanisms by which HCC modifies the homeostatic balance to lean toward hypercoagulability have been proposed. One study suggested that tissue factor (TF), the initiator of the coagulation process, was related to angiogenesis and invasiveness of HCC, and an elevated level of TF was an independent prognostic factor in HCC patients (29). Another study showed that circulating microparticles (MP), a population of extracellular vesicles, have the ability to induce coagulation and promote portal vein thrombosis (PVT) in patients with concomitant cirrhosis and HCC, possibly due to higher MP TF activity in these patients (30).

Several limitations of this study have to be acknowledged. First, this is a retrospective cohort study with its intrinsic bias. Nonetheless, the large sample size from six high-volume hospitals increases its reliability. Second, majority of the study participants had a history of HBV infections. Future studies involving patients with hepatitis C virus infections or alcoholism as the primary etiological factors of HCC are needed. Last, the underlying biological mechanisms between hypercoagulability and PVTT formation and progression have not been explored in this study.

In conclusion, there is a close association between preoperative INR level and the incidence and extent of PVTT in HCC patients. HCC patients with hypercoagulability in the Low INR group had a significantly higher incidence of PVTT and more extensive PVTT. HCC patients with PVTT limited to a first-order branch or above of the main portal vein in the Low INR group had worse survival outcomes than those in the Normal and High INR groups after R0 LR.

## Data Availability Statement

The raw data supporting the conclusions of this article will be made available by the authors, without undue reservation.

## Ethics Statement

The studies involving human participants were reviewed and approved by Eastern Hepatobiliary Surgery Hospital Foshan First People’s Hospital Fujian Cancer Hospital the Affiliated Hospital of Binzhou Medical College Wenzhou People’s Hospital LongYan First Hospital. The patients/participants provided their written informed consent to participate in this study.

## Author Contributions

Conception and design: S-QC, WL, M-CW, and X-PZ. Financial support: S-QC. Provision of study materials or patients: Z-JZ, DZ, FZ, Y-RH, C-QZ, Z-TC, KW, JS, and W-XG. Collection and assembly of data: X-PZ, T-FZ, J-KF, Z-YS, and Z-HC. Data Analysis and interpretation: X-PZ, T-FZ, J-KF, and Z-YS. All authors contributed to the article and approved the submitted version.

## Funding

This work was supported by the Key Project of the National Natural Science Foundation of China (No: 81730097), the Grants of the Science Fund for Creative Research Groups (No: 81521091), the National Natural Science Foundation of China (No: 82072618), and Shanghai Municipal Health Bureau (No: SHDC2020CR1004A).

## Conflict of Interest

The authors declare that the research was conducted in the absence of any commercial or financial relationships that could be construed as a potential conflict of interest.

## Publisher’s Note

All claims expressed in this article are solely those of the authors and do not necessarily represent those of their affiliated organizations, or those of the publisher, the editors and the reviewers. Any product that may be evaluated in this article, or claim that may be made by its manufacturer, is not guaranteed or endorsed by the publisher.

## Supplementary Material

Supplementary Figure 1 Kaplan-Meier analysis for subgroup analysis of the RFS and OS rates in HCC patients with types I/II PVTT after R0 LR among the Low, Normal, and High INR groups. RFS for patients with type I PVTT among the Low, Normal, and High INR groups (55 patients *vs* 113 patients *vs* 37 patients) after R0 LR **(A)** (P < 0.001); OS for patients with type I PVTT among the Low, Normal, and High INR groups (55 patients *vs* 113 patients *vs* 37 patients) after R0 LR **(B)** (P < 0.001); RFS for patients with type II PVTT among the Low, Normal, and High INR groups (89 patients *vs* 229 patients *vs* 69 patients) after R0 LR **(C)** (P = 0.019); OS for patients with type II PVTT among the Low, Normal, and High INR group (89 patients *vs* 229 patients *vs* 69 patients) after R0 LR **(D)** (P < 0.001). INR, international normalized ratio.
