# Supplementary figures and images for: Association of Preoperative Coagulability With Incidence and Extent of Portal Vein Tumor Thrombus and Survival Outcomes in Hepatocellular Carcinoma After Hepatectomy: A Large-Scale, Multicenter Study
Source: Front Oncol. 2021 Jul 28;11:697073. doi: 10.3389/fonc.2021.697073 (PMC8356674; doi:10.3389/fonc.2021.697073)

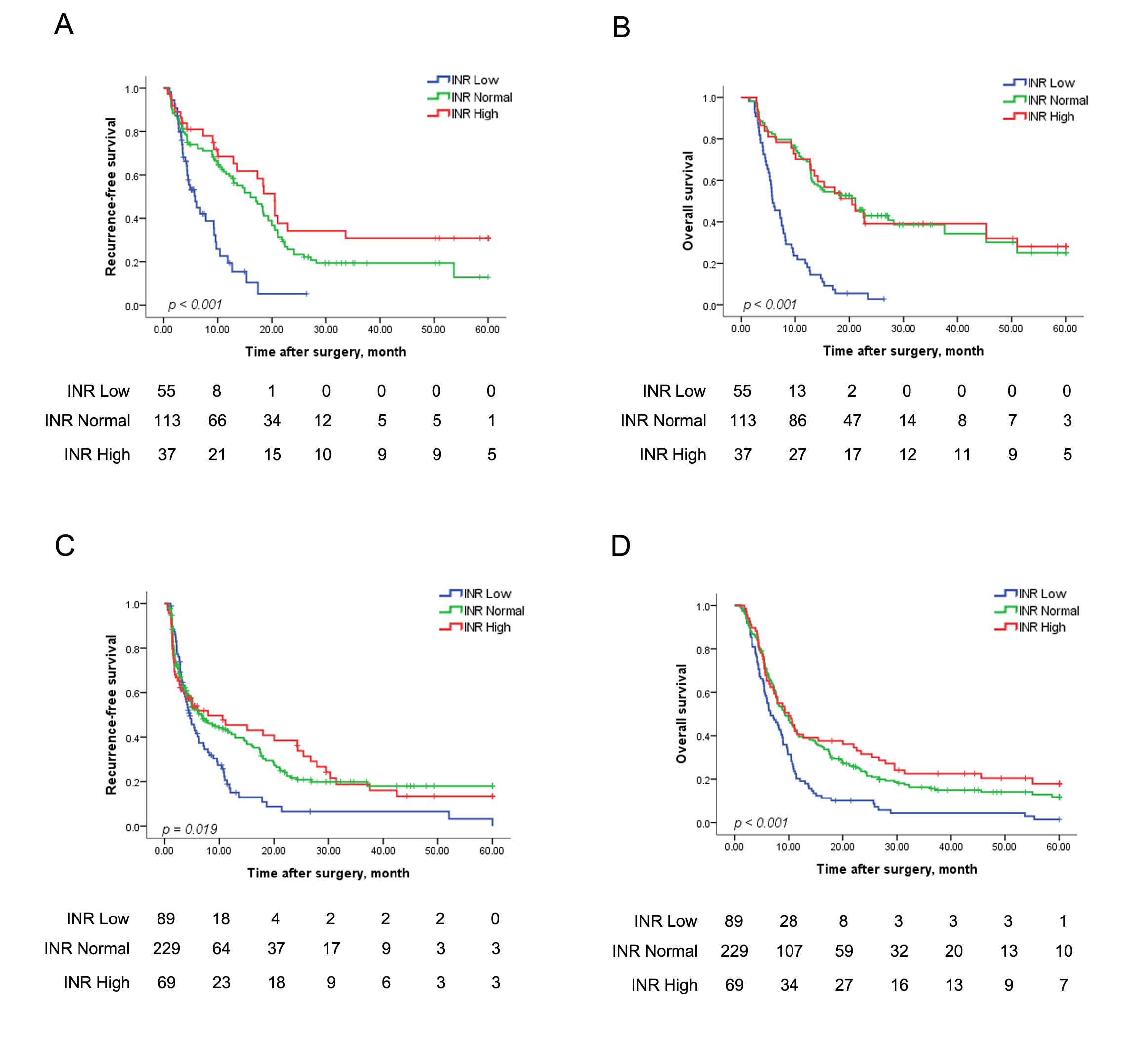

Supplement: Supplementary Figure 1 — Kaplan-Meier analysis for subgroup analysis of the RFS and OS rates in HCC patients with types I/II PVTT after R0 LR among the Low, Normal, and High INR groups. RFS for patients with type I PVTT among the Low, Normal, and High INR groups (55 patients vs 113 patients vs 37 patients) after R0 LR (A) (P < 0.001); OS for patients with type I PVTT among the Low, Normal, and High INR groups (55 patients vs 113 patients vs 37 patients) after R0 LR (B) (P < 0.001); RFS for patients with type II PVTT among the Low, Normal, and High INR groups (89 patients vs 229 patients vs 69 patients) after R0 LR (C) (P = 0.019); OS for patients with type II PVTT among the Low, Normal, and High INR group (89 patients vs 229 patients vs 69 patients) after R0 LR (D) (P < 0.001). INR, international normalized ratio. [file Image_1.tif]
